# Seratrodast inhibits ferroptosis by suppressing lipid peroxidation

**DOI:** 10.1038/s41419-024-07251-y

**Published:** 2024-11-22

**Authors:** Juliane Tschuck, Wulf Tonnus, Shubhangi Gavali, Andrea Kolak, Melodie Mallais, Francesca Maremonti, Mami Sato, Ina Rothenaigner, José Pedro Friedmann Angeli, Derek A. Pratt, Andreas Linkermann, Kamyar Hadian

**Affiliations:** 1 Research Unit Signaling and Translation, Helmholtz Zentrum München, Neuherberg, Germany; 2 Division of Nephrology, Department of Internal Medicine 3, University Hospital Carl Gustav Carus at the Technische Universität Dresden, Dresden, Germany; 3 Department of Medicine V, University Medical Centre Mannheim, University of Heidelberg, Mannheim, Germany; 4 Department of Chemistry and Biomolecular Sciences, University of Ottawa, Ottawa, ON Canada; 5 Rudolf Virchow Center for Integrative and Translational Bioimaging, Chair of Translational Cell Biology, University of Würzburg, Würzburg, Germany; 6 Division of Nephrology, Department of Medicine, Albert Einstein College of Medicine, Bronx, NY USA

**Keywords:** Cell death, Acute kidney injury, Drug development

## Abstract

Ferroptosis is a regulated and non-apoptotic form of cell death mediated by iron-dependent peroxidation of polyunsaturated fatty acyl tails in phospholipids. Research of the past years has shed light on the occurrence of ferroptosis in organ injury and degenerative diseases of the brain, kidney, heart, and other tissues. Hence, ferroptosis inhibition may prove therapeutically beneficial to treat distinct diseases. In this study, we explored the ferroptosis-modulating activity of seratrodast, an inhibitor of thromboxane A2 (TXA2) receptor, which is approved in some countries for the treatment of asthma. Interestingly, seratrodast suppressed ferroptosis, but not apoptosis and necroptosis; thus, demonstrating selective anti-ferroptotic activity. While seratrodast itself does not inhibit lipid peroxidation, it exhibits potent radical-trapping antioxidant activity upon reduction to its corresponding hydroquinone form—analogously to ubiquinone and vitamin K. Importantly, seratrodast ameliorated the severity of renal ischemia-reperfusion injury in mice. Together, this study provides a drug repurposing case, where seratrodast—a marketed drug—can undergo fast-forward preclinical/clinical development for the inhibition of ferroptosis in distinct degenerative diseases.

## Introduction

Ferroptotic cell death, a distinct regulated non-apoptotic cell death modality, is characterized by extensive iron-dependent lipid peroxidation [1, 2], which can be the cause for distinct degenerative diseases, such as neurodegeneration or kidney degeneration. Moreover, tissue or organ damage including ischemia-reperfusion injury (IRI), acute kidney failure, cardiomyopathy, or stroke may be caused by ferroptosis [3–5]. To prevent ferroptosis, cells have developed manifold ferroptosis-inhibitory mechanisms [4, 6]. The major gatekeeper system is the system x_C_^−^/glutathione/GPX4 axis [4, 7], which suppresses lipid peroxidation by reducing lipid peroxides to their non-lethal alcohol forms and thereby counteracting ferroptotic cell death. In addition, a number of other effective anti-ferroptotic pathways have been discovered that act independently from the system x_C_^-^/glutathione/GPX4 axis: these are mainly the FSP1-ubiquinol axis [8, 9], the GCH1/tetrahydrobiopterin/DHFR axis [10, 11] and 7-Dehydrocholestrol (7-DHC) [12, 13]. These inhibitory pathways generally involve the synthesis or regeneration of small-molecule radical-trapping antioxidants (RTAs) [14]. More recently, FSP1/vitamin K [15] as well as hydropersulfides [16, 17] have been shown to inhibit ferroptosis. Moreover, agonistic activation of the farnesoid X receptor (FXR) by bile acids as well as the retinoic acid receptor (RAR) by vitamin A can act as master regulators to upregulate key ferroptosis gatekeepers counteracting lipid peroxidation and ferroptosis [18, 19].

Ferroptosis can be chemically triggered by inhibiting the cystine-glutamate antiporter system x_C_^−^ *e.g*., by erastin or imidazole ketone erastin (IKE), or by directly inhibiting glutathione peroxidase 4 (GPX4) using *e.g*., (1*S*,3*R*)-RSL3 and ML210. Additionally, FIN56 as well as FINO_2_ can execute ferroptosis by mixed effects [6]. All these molecules mainly inhibit the system x_C_^−^/glutathione/GPX4 axis to limit protection against lipid peroxidation. In contrast, numerous small molecule RTAs such as ferrostatin-1, liproxstatin-1, and vitamin E are capable of suppressing lipid peroxidation [4, 6, 20], thereby inhibiting ferroptosis. Despite the availability of a number of ferroptosis-modulating compounds, most of them are either not in vivo compatible or still in preclinical development [3]; thus, raising the need for additional strategies to develop ferroptosis-based medicines [21, 22]. Drug repurposing of clinical-stage or approved molecules can be an efficient way to fast-track preclinical drug development of advanced candidates towards clinical application [23].

In this study, we explored whether seratrodast could be repurposed for ferroptosis inhibition. Seratrodast is a thromboxane A2 (TXA2) receptor antagonist and approved for the treatment of asthma in Japan, China and India [24, 25]. We demonstrate specific suppression of ferroptosis by seratrodast and show that the radical-trapping antioxidant activity of seratrodast, once reduced to its hydroquinone form, counteracts lipid peroxidation. In line with this, seratrodast reduces severity of acute kidney injury in mice. Therefore, seratrodast may be a candidate for fast-track clinical application to alleviate ferroptosis-based organ damage or degenerative diseases.

## Results

### Seratrodast inhibits ferroptosis

**Fig. 1:**
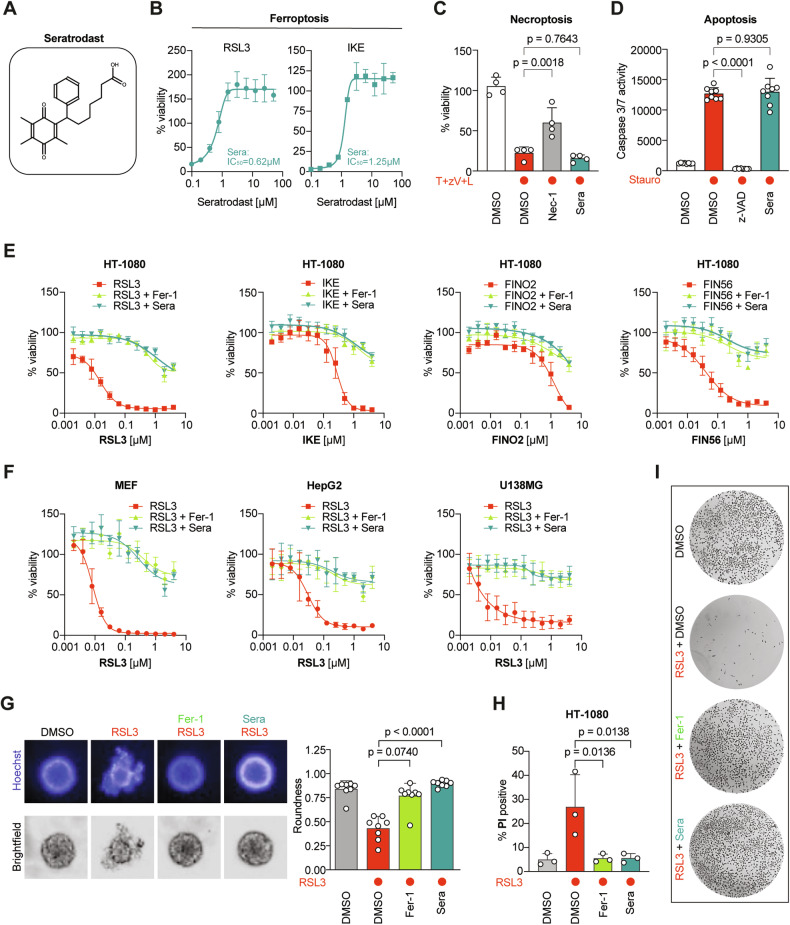
Seratrodast specifically inhibits ferroptosis in monolayer and 3D cell models. **A** Chemical structure of seratrodast. **B** Seratrodast (Sera) dose-dependently suppresses ferroptosis induced by 100 nM RSL3 or 1.5 µM IKE for 18 h. Data plotted are mean ± SD (*n* = 4 biological replicates). **C** Seratrodast does not inhibit necroptosis. T+zV+L = 20 ng/ml TNFα + 10 µM Z-VAD-FMK + 10 µM LCL161 for 18 h; 10 µM necrostatin-1 (Nec-1); 6 µM seratrodast; data plotted are mean ± SD (*n* = 4 biological replicates); One-way ANOVA with Dunnett’s test. **D** Seratrodast does not inhibit apoptosis. 1 µM staurosporine (Stauro); 50 µM Z-VAD-FMK; 6 µM seratrodast for 18 h; data plotted are mean ± SD (*n* = 3 biological replicates); Dunnett’s T3 multiple comparisons test. **E** Seratrodast inhibits ferroptosis induced by RSL3, IKE, FINO2 and FIN56, comparable to ferrostatin-1. 2 µM Fer-1; 6 µM seratrodast for 18 h. Cell viability was normalized to DMSO-treated cells. Data plotted are mean ± SEM (*n* = 4 biological replicates). **F** Seratrodast inhibits ferroptosis induced by RSL3 in different cell lines. 2 µM Fer-1; 6 µM seratrodast for 18 h. Cell viability was normalized to DMSO-treated cells. Data plotted are mean ± SD (*n* = 3 biological replicates). **G** Seratrodast inhibits ferroptosis in a 3D spheroid model. 200 nM RSL3; 2 µM Fer-1; 6 µM seratrodast for 48 h. Representative image of *n* = 8 spheroids per condition is shown; spheroid roundness was quantified using high-content image analysis; data plotted are mean ± SD (*n* = 8), Kruskal–Wallis test with Dunn’s multiple comparisons. **H** Live-dead discrimination via PI staining and flow cytometry of HT-1080 treated with 200 nM RSL3, 2 µM Fer-1 and 6 µM seratrodast for 2.5 h. Data plotted are mean ± SD (*n* = 3 biological replicates), One-way ANOVA with Dunnett’s test. **I** Crystal-violet staining of HT-1080 cells treated with 200 nM RSL3, 2 µM Fer-1 and 6 µM seratrodast as indicated for 24 h.

To pursue a drug repurposing approach for ferroptosis inhibition, we rationally looked at previously clinically-applied small molecules with potential RTA activity. Here, we studied seratrodast (Fig. 1A), a thromboxane A2 receptor (TXA2) antagonist [25], which contains a redox-active quinone moiety. Notably, ubiquinol [9] and vitamin K [15] also contain a *para*-quinone structure, which is reduced in an FSP1-dependent manner to their corresponding *para*-hydroquinones, a functionality with good radical-trapping antioxidant activity and therefore anti-ferroptotic feature. Therefore, we investigated whether seratrodast could also inhibit ferroptosis in HT-1080 cells, a well-established model cell line in the ferroptosis field. We tested effects of seratrodast on different cell death modalities using cellular viability assays. Seratrodast effectively suppressed ferroptosis induced by RSL3 or IKE (Fig. 1B), but not necroptosis (Fig. 1C) and apoptosis (Fig. 1D); hence, demonstrating selective capability to inhibit ferroptosis over the other regulated cell death pathways.

Next, we used various ferroptosis inducers (FINs) to understand if seratrodast universally counteracts ferroptosis. To this end, we induced ferroptosis with IKE (class I FIN – inhibitor of system x_C_^-^), RSL3 (class II FIN – inhibitor of GPX4), FIN56, and FINO_2_. Seratrodast was able to suppress ferroptosis induced by all four FINs (Fig. 1E), and was in the same efficacy range as ferrostatin-1 [6], a reference ferroptosis inhibitor. The protective effect of seratrodast against RSL3-induced ferroptosis was reproduced in additional cell lines, *i.e*., mouse embryonic fibroblasts (MEF), hepatocellular carcinoma (HepG2) and glioblastoma (U138MG) (Fig. 1F). We further analyzed if seratrodast would inhibit ferroptotic cell death in a 3D spheroid model system (clustered 3D cell culture) that encompasses a more physiological setting rather than 2D monolayer culture. Treatment of HT-1080-derived spheroids with RSL3 led to dissociation of the 3D organization, which was quantified by reduction of roundness (Fig. 1G). Importantly, ferrostatin-1 as well as seratrodast fully inhibited ferroptosis-based spheroid destruction (Fig. 1G). We further quantified cell death by staining HT-1080 cells with propidium iodide (PI) after ferroptosis induction via RSL3. Analysis by flow cytometry showed a reduction of PI-positive cells in cell populations co-treated with seratrodast, similar to the positive control ferrostatin-1 (Fig. 1H, Supplementary Fig. 1). This result was also visualized when HT-1080s were fixed and stained with crystal violet (Fig. 1I).

### Seratrodast is a radical-trapping antioxidant and inhibits lipid peroxidation

**Fig. 2:**
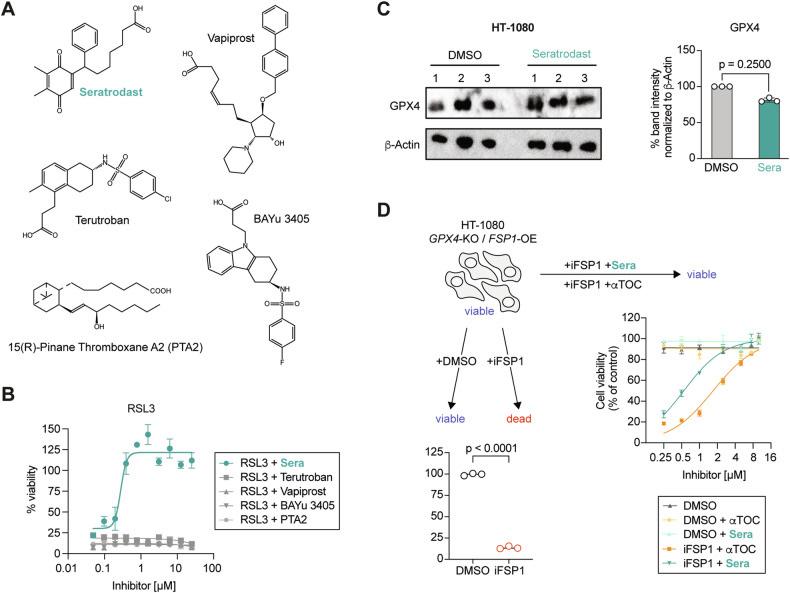
Seratrodast, but not other TXA2 receptor inhibitors suppress ferroptosis. **A** Structures of different TXA2 receptor antagonists. Only seratrodast contains a quinone moiety. **B** TXA2 receptor antagonists that lack a quinone moiety are not able to inhibit ferroptosis. 200 nM RSL3 and indicated antagonist concentration range for 18 h; data plotted are mean ± SD (n = 3 biological replicates). **C** Western Blot of HT-1080 treated with DMSO or 6 µM seratrodast for 6 h. GPX4 band intensity was normalized to β-Actin signal. Data plotted are mean ± SD (*n* = 3 biological replicates), Wilcoxon matched-pairs signed rank test. **D** HT-1080 *GPX4*-KO *FSP1*-OE treated with iFSP1 to kill cells (*n* = 3 technical replicates), Welch’s unpaired *t*-test. Seratrodast (Sera) and α-tocopherol (αTOC) dose-dependently inhibit iFSP1-induced ferroptosis in HT-1080 *GPX4*-KO *FSP1*-OE cells.

To understand the mechanism behind the ferroptosis-inhibitory potential of seratrodast and to experimentally uncouple a potential radical-trapping antioxidant activity from TXA2 receptor inhibition as the cause of ferroptosis inhibition, we tested four additional TXA2 receptor antagonists (terutroban, vapiprost, BAYu 3405, and 15(R)-pinane thromboxane A_2_ (PTA2)) – all these molecules lacking a quinone moiety (Fig. 2A), in contrast to seratrodast. Interestingly, all four other TXA2 receptor inhibitors were unable to suppress ferroptotic cell death. Only seratrodast with the quinone structure suppressed ferroptosis in the same setting; thus, we hypothesized that the radical-trapping antioxidant activity of the (reduced) quinone moiety of seratrodast was responsible for inhibition of ferroptosis (Fig. 2B). To explore a possible effect of seratrodast on the key ferroptosis-inhibitory enzyme GPX4 as recently suggested [26], we treated HT-1080 cells with seratrodast and investigated GPX4 protein levels by Western Blot analysis (Fig. 2C). No changes in GPX4 abundance in seratrodast-treated cells compared to DMSO-treated control cells were observed, which further supports our hypothesis of a sole antioxidant effect of seratrodast. To unequivocally prove that seratrodast has no effect on GPX4 levels, we used HT-1080 *GPX4*-KO *FSP1*-OE cells that are viable but die once treated with an FSP1 inhibitor (iFSP1) (Fig. 2D). Importantly, dose-dependent treatment with seratrodast or α-tocopherol (a well-known ferroptosis inhibitor) can completely prevent iFSP1-induced ferroptosis in HT-1080 *GPX4*-KO *FSP1*-OE cells, demonstrating a GPX4-independent role of seratrodast in ferroptosis inhibition (Fig. 2D).

**Fig. 3:**
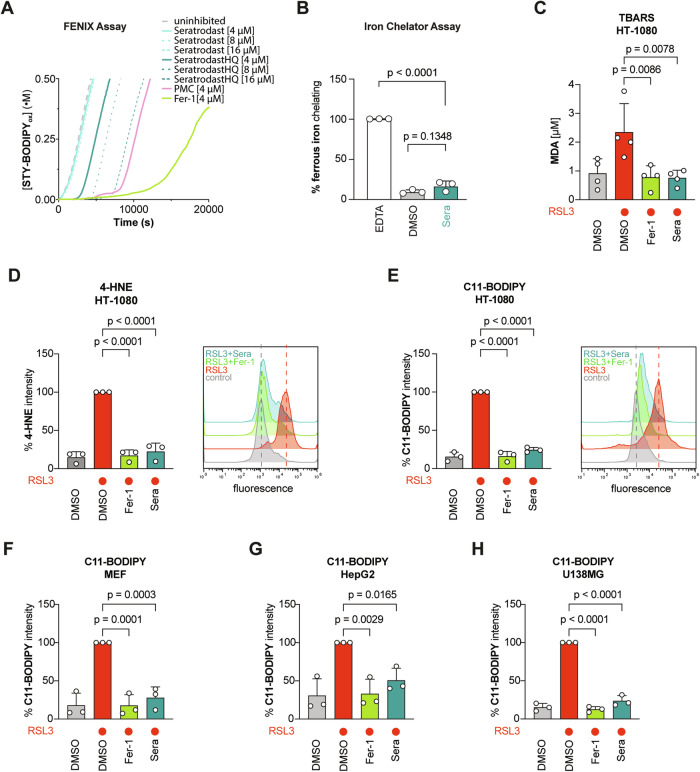
Seratrodast inhibits ferroptosis by reducing lipid peroxidation. **A** Representative co-autoxidation of STY-BODIPY (1 µM) and the polyunsaturated lipids of liposomes egg phosphatidylcholine (1 mM, 100 nm particles) initiated by di-tert-undecylhyponitrite (200 µM) and inhibited by 4, 8 and 16 µM of either seratrodast or its corresponding hydroquinone, or 4 µM of either ferrostatin-1 or 2,2,5,7,8-pentamethylchroman-6-ol (PMC). Quantitative analyses and statistics for multiple replicates are given in Supplementary Fig. 2. **B** Iron chelator assay with seratrodast and EDTA as an assay control. Seratrodast has no iron-chelating activity; data plotted are mean ± SD (*n* = 3 biological replicates); One-Way ANOVA with Tukey’s test. **C** Seratrodast reduces RSL3-induced malondialdehyde (MDA)—a product of lipid peroxidation. 250 nM RSL3; 2 µM Fer-1; 6 µM seratrodast for 2.5 h; data plotted are mean ± SD (*n* = 3 biological replicates); One-way ANOVA with Dunnett’s test. **D** Seratrodast reduces RSL3-induced 4-hydroxynonenal (4-HNE) — a product of lipid peroxidation in HT-1080 cells. 250 nM RSL3; 2 µM Fer-1; 6 µM seratrodast for 2.5 h; data plotted are mean ± SD (*n* = 3 biological replicates); One-way ANOVA with Dunnett’s test. Representative histogram is shown. **E**–**H** Seratrodast limits RSL3-mediated oxidation of the lipid peroxidation sensor C11-BODIPY. (**E**) C11-BODIPY measurement in HT-1080. 200 nM RSL3; 2 µM Fer-1; 6 µM seratrodast for 2.5 h. Representative histogram is shown. Quantification of *n* = 3 biological replicates is depicted; data plotted are mean ± SD (*n* = 3); One-way ANOVA with Dunnett’s test. For validation purposes, C11-BODIPY staining was repeated in the following cell lines: MEF cells (**F**), HepG2 cells (**G**), and U138MG cells (**H**).

To demonstrate the radical-trapping antioxidant activity of seratrodast, we carried out FENIX lipid peroxidation assays [27]. These cell-free assays utilize the fluorescent peroxidation-sensitive reporter STY-BODIPY [28] to report on the rate of lipid peroxidation and its inhibition by added RTAs in liposomes of phosphatidylcholine lipids. The results make clear that while seratrodast itself is not an inhibitor, its corresponding hydroquinone (independently prepared by catalytic hydrogenation) is a potent RTA (Fig. 3A, Supplementary Fig. 2). The rate of STY-BODIPY oxidation during the inhibited period corresponds to a rate constant for reaction with (phospho)lipid peroxyl radicals of *k*_inh_ = 4.2 × 10^4^ M^−1^ s^−1^, which is slightly lower than that determined for ferrostatin-1 (*k*_inh_ = 6.6 × 10^4^ M^−1^ s^−1^), and similar to 2,2,5,7,8-pentamethylchroman-6-ol (PMC), the reactive headgroup of α-tocopherol, the most active form of Vitamin E (*k*_inh_ = 3.7 × 10^4^ M^−1^ s^−1^). The lower radical-trapping stoichiometry (*n* = 0.5) than ferrostatin-1 (*n* = 3.0) and PMC (*n* = 2), presumably results from the hydroquinone’s lability to adventitious oxidation [29], a reality shared by the active (reduced) forms of related ubiquinone and the vitamin K derivatives [9, 15].

We also investigated whether seratrodast may have iron-chelating activity and inhibits ferroptosis via this mechanism, however, this could be ruled out, since seratrodast showed no capacity to chelate ferrous iron (Fig. 3B). Furthermore, seratrodast reduced levels of malondialdehyde (MDA)—a cellular marker of lipid peroxidation—induced upon RSL3 treatment in TBARS assays (Fig. 3C). Similarly, 4-hydroxynonenal (4-HNE) levels—aldehyde products of lipid peroxidation non-enzymatically generated upon RSL3 treatment – were also significantly reduced by seratrodast (Fig. 3D). Finally, the level of RSL3-mediated peroxidation of the C11-BODIPY sensor in HT-1080 cells was largely reduced upon seratrodast treatment (Fig. 3E), and this effect was reproduced in MEFs, HepG2, and U138MG cells (Fig. 3F–H). All data show that seratrodast effectively reduces lipid peroxidation in cell-free settings as well as in cells. Notably, and consistent with the results of the FENIX assay, all ferroptosis-inhibitory effects of seratrodast as well as its effect on lipid peroxidation were comparable to, but systematically lower than, the reference molecule ferrostatin-1.

### Seratrodast protects from renal ischemia-reperfusion injury in mice

**Fig. 4:**
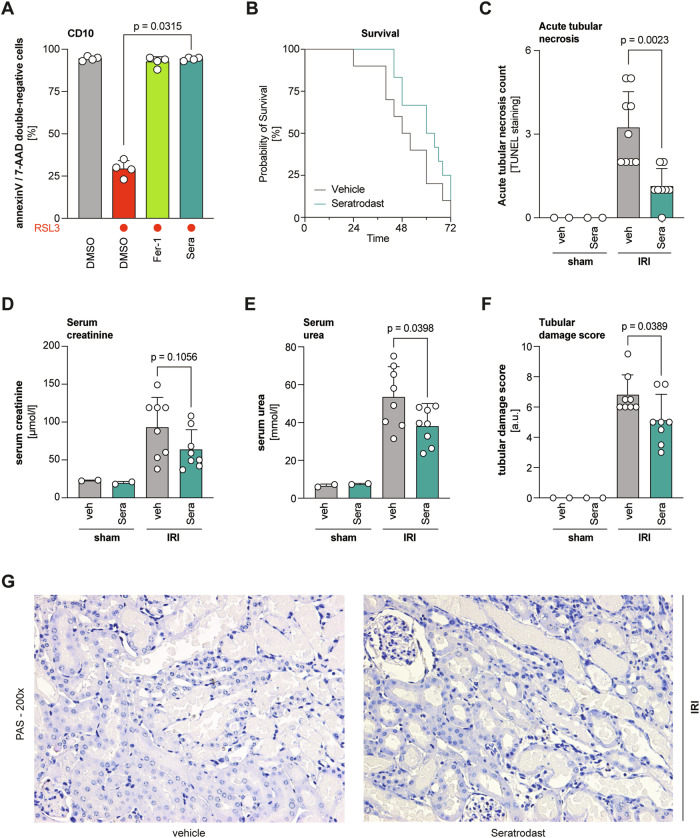
Seratrodast protects from renal ischemia-reperfusion injury in mice. **A** Seratrodast protects human kidney tubular cells from RSL3-induced ferroptosis in an annexin V/7-AAD staining. 1.13 µM RSL3, 1 µM Fer-1, 6 µM seratrodast for 6 h. Data shown are mean ± SD (*n* = 4 independent biological replicates); Kruskal–Wallis with Dunn’s test. **B** Seratrodast treatment demonstrated a trend to improved survival probability of mice upon lethal IRI (median survival improved from 50 h to 62 h). *n* = 12 mice per condition, Logrank (Mantel–Cox) test and Gehan–Breslow–Wilcoxon test were performed. **C** TUNEL staining was performed in IRI-induced mice and showed less acute tubular necrosis in seratrodast-treated mice. Data plotted is mean ± SD of *n* = 8–9 samples/group; Kolmogorov-Smirnov test. **D**–**G** Male C57Bl/6N mice were injected intraperitoneally with a single dose of 15 mg/kg seratrodast or vehicle 30 min before IRI was induced by clamping renal arteries. Forty-eight hours after surgery, blood was taken via retroorbital puncture and analyzed for different markers of kidney functionality. Seratrodast injections lead to reduced levels of serum creatinine (**D**) and serum urea (**E**). Data plotted are mean ± SD (*n* = 8 mice/group); unpaired t-test with Welch’s correction. **F** Seratrodast treatment reduced the tubular damage score after IRI. Data plotted are mean ± SD (*n* = 8 mice/group); unpaired *t*-test with Welch’s correction. **G** Kidneys of mice that underwent IRI surgery were stained with Periodic acid–Schiff staining (PAS) and assessed for morphological damage in a double-blind manner. Representative images are shown.

Since the involvement of ferroptosis in acute kidney injury is well described [30, 31], we tested seratrodast in the human kidney tubular cell line CD10-135. Upon ferroptosis induction with RSL3, cells were co-treated with seratrodast or ferrostatin-1 and co-stained with annexin V and 7-AAD. Flow cytometry data showed a strong rescue effect of seratrodast to prevent ferroptotic cell death (Fig. 4A, Supplementary Fig. 3A). Moreover, seratrodast reduced lipid peroxidation in CD10-135 as determined by C11-BODIPY staining (Supplementary Fig. 3B). To translate our findings into a clinically relevant disease model, we investigated the effect of seratrodast on murine renal ischemia/reperfusion injury. This disease model is widely used for in vivo ferroptosis research and already successfully yielded important insights into the pathophysiological role of ferroptosis [31, 32]. Following a lethal dose of ischemia, seratrodast-treated mice demonstrated a trend toward prolonged survival compared to vehicle-treated ones (Fig. 4B). In a second experimental setup, healthy 10-week-old male mice were injected with either vehicle or 15 mg/kg seratrodast in a single dose intraperitoneally, before IRI was induced by clamping both renal pedicles. As a control group, mice were sham operated without the induction of IRI. After 48 h, mice were sacrificed and renal injury analyzed. TUNEL staining of kidney slices was performed to quantify acute tubular necrosis following IRI induction (Supplementary Fig. 4A). Importantly, seratrodast-treated mice showed significantly less tubular necrosis than vehicle-treated mice (Fig. 4C). Furthermore, seratrodast-treated mice showed lower levels of serum creatinine and serum urea (Fig. 4D, E), indicating that the treatment alleviated IRI-induced kidney damage. To confirm these findings, kidney slices were stained with Periodic acid–Schiff reagent (PAS), imaged, and scored according to morphological features such as brush border loss, tubule dilatation or tubular cast formation (Fig. 4F, G, Supplementary Fig. 4B). Notably, the tubular damage of seratrodast-treated mice was mostly ranked at stage 3–6 (mild to moderate damage), whereas vehicle-treated mice were ranked at stage 6–9 (severe damage), further supporting our repurposing hypothesis of seratrodast as a clinical candidate against ferroptosis-caused tissue damage.

## Discussion

There is a growing body of evidence that ferroptosis is a key cell death modality in degenerative diseases and organ injury [2, 4]. Thus, there is a great need to generate ferroptosis-based medicines for clinical testing [21, 22]. The past decade has fueled the identification and development of a number of small-molecule ferroptosis inhibitors, but none of them is currently at the stage of clinical testing [2, 3]. A recent drug repurposing screen by the Dixon lab suggested that the FDA-approved drug bazedoxifene is a radical-trapping antioxidant, which inhibits ferroptosis [33]. In this report, we present another case of drug repurposing that may bridge the gap to clinical application of ferroptosis by introducing the FDA-approved drug seratrodast as a radical-trapping antioxidant and a potent ferroptosis inhibitor. Seratrodast was originally developed as a thromboxane A2 (TXA2) receptor antagonist and marketed in some countries (Japan, China, and India) to treat asthma with mild to moderate side effects [24]. A very recent study suggests that seratrodast inhibits ferroptosis by elevating GPX4 levels through modulating the TXA2 receptor [26]. In our report, however, we reveal that the reduced (hydroquinone) form of seratrodast has radical-trapping antioxidant activity, and this is sufficient to revert lipid peroxidation and suppress ferroptosis. We do not see an impact of TXA2 receptor inhibition on ferroptosis suppression, as several other TXA2 receptor antagonists without a quinone structure failed to inhibit ferroptosis. In contrast, we demonstrate that the chemical structure of seratrodast comprises a quinone moiety which, once reduced, affords a potent radical-trapping antioxidant function analogous to that found in ubiquinol and vitamin K [15]. Moreover, we show that levels of GPX4 are unaffected upon seratrodast treatment and that seratrodast fully inhibits ferroptosis independent of GPX4 in HT-1080 *GPX4*-KO cells. Interestingly, some antioxidant activity was previously linked to seratrodast [34].

We could show that seratrodast treatment alleviated markers of acute kidney injury (AKI) in mice, which indicates that this drug could be suitable to counteract ferroptosis in relevant pathophysiological cases in patients. Interestingly, also other studies have shown that repurposing molecules, such as cytochrome P450 substrate drugs, can have radical scavenging activity and suppress organ damage by AKI [35]. Notably, we anticipate that seratrodast will have a better safety profile compared to cytochrome P450 substrate drugs. In future experiments, other ferroptosis-related in vivo models of tissue damage, such as myocardial infarction, could be employed to evaluate the therapeutic potential of seratrodast. Ferroptotic cell death was also found to contribute to pulmonary diseases, such as asthma, COPD, lung fibrosis or COVID-19 [36, 37]. Since seratrodast was originally developed as a TXA2 antagonist to inhibit vasoconstriction during asthma, and asthma patients show elevated levels of iron and lipid peroxidation [38], it is interesting to explore if seratrodast improves asthma in patients by inhibiting ferroptosis.

Together, in this study, we provide evidence that seratrodast, a marketed drug antagonizing thromboxane A2 (TXA2) receptor, reduces lipid peroxidation and suppresses ferroptosis via its quinone moiety in cellular models and a kidney IRI preclinical mouse model. Hence, seratrodast may represent a valuable drug repurposing case to treat ferroptosis-based organ damage.

## Methods

### Cell culture

Human fibrosarcoma cell line HT-1080, immortalized mouse embryonic fibroblasts (MEF), human hepatocyte carcinoma cell line HepG2, human glioblastoma cell line U138MG and immortalized human renal tubular cell line CD10-135 were used. MEFs were a gift from Daniel Krappmann (Helmholtz Munich), HT-1080, HepG2 and U138MG were purchased from ATCC, and CD10-135 were originally generated and provided by Rafael Kramann (RWTH Aachen) as described previously [31]. All cell lines were grown in Dulbecco’s Modified Eagle’s medium (Thermo Fisher Scientific, 41966-029) supplemented with 10% fetal bovine serum (FBS, Thermo Fisher Scientific), 1% penicillin–streptomycin (Thermo Fisher Scientific) and 1% non-essential amino acids (MEM NEAA, Thermo Fisher Scientific). Cells were grown at 37 °C and 5% CO_2_.

### Compounds

For ferroptosis assays, (1S,3R)-RSL3 (RSL3, Sigma), imidazole ketone erastin (IKE, Cayman Chemical), FIN56 (Cayman Chemical), FINO_2_ (Cayman Chemical), seratrodast (Sera, Cayman Chemical) and ferrostatin-1 (Fer-1, Sigma) were purchased. For the apoptosis and necroptosis assay, staurosporine (Stauro, TargetMol) and Z-VAD-FMK (zVAD, TargetMol), LCL161 (MedChemExpress), tumor necrosis factor alpha (TNFα, biomol) and necrostatin-1 (Nec-1, BioVision) were purchased. Following TXA2 receptor antagonists were purchased: terutroban (Sigma), vapiprost (Santa Cruz Biotechnology), ramatroban (BAYu 3405, Santa Cruz Biotechnology) and 15(R)-pinane thromboxane A_2_ (PTA2, Cayman Chemical).

### Cell viability assays

HT-1080 were seeded into 384-well plates (CulturPlates, PerkinElmer) with a density of 750 cells per well. After 24 h of growth, cells were treated with 10 or 12-point serial dilutions of compounds in indicated concentrations for 18 h. Viability was detected by using CellTiter-Glo® 2.0 Reagent (Promega) according to the manufacturer’s instruction and luminescence was read out in an EnVision 2104 Multilabel plate reader (PerkinElmer).

For detecting apoptosis, HT-1080 were treated with staurosporine before addition of seratrodast or Z-VAD-FMK. Caspase 3/7 activity was measured 18 h later via Caspase-Glo® 3/7 Assay Reagent (Promega) according to the manufacturer’s instruction. Cells were incubated for 45 min at room temperature and luminescence was measured. For detection of necroptosis, MEF were treated with TNFα, Z-VAD-FMK and LCL161 before addition of seratrodast or necrostatin-1. Cell viability was measured after 18 h by adding CellTiter-Glo® 2.0 Reagent (Promega) according to the manufacturer’s instruction and luminescence was read out.

For evaluating the cytoprotective effect of seratrodast upon FSP1 inhibition in *GPX4*-knockout (KO)/*FSP1*-overexpression (OE) cells, HT-1080 *GPX4*-KO/ *FSP1*-OE were plated at 3000 cells/ well in a 96-well plate 4 h ahead of the treatment and co-treated with 3 µM FSP1 inhibitor (iFSP1) and seratrodast or α-tocopherol with different concentrations. After 24 h incubation, cell viability was assessed using Alamar Blue. Following the manufacturer’s instruction, cells were incubated in resazurin solution diluted 1:100 with a standard cell culture medium for 2–4 h. Fluorescence at Ex: 540 nm/ Em: 590 nm was measured in Spark® microplate reader (Tecan).

### Spheroid formation and imaging

2,000 HT-1080 cells were seeded per well into a 96-well Round Bottom Ultra Low Attachment Microplate (Corning costar 7007). Over a period of 48 h spheroids were formed and subsequently treated with RSL3, and ferrostatin-1 or seratrodast. After another 48 h, spheroids were stained using Hoechst 33342 (Sigma) in a 1:10,000 dilution and incubated for 1 h. Images were acquired using an Operetta high-content imaging system (PerkinElmer) and analyses were conducted with the Columbus software (PerkinElmer). For analysis, spheroids were detected as “image region” and morphology properties were calculated (roundness).

### FENIX lipid peroxidation assay

Liposomes (1.03 mM) prepared from egg phosphatidylcholine [39] and STY-BODIPY (1.03 µM) were combined in chelex-treated phosphate-buffered saline (cPBS) (12 mM phosphate, 150 mM NaCl, pH 7.4) and added to the wells of a Nunc black polypropylene round-bottomed 96-well microplate (290 µL). Using a 1–10 µL multichannel pipette, 5 µL of inhibitor stock solution or vehicle (DMSO) were added to the wells and manually mixed using a 100–300 µL multichannel pipette (set to 250 µL). The microplate was then inserted into a BioTek H1 synergy microplate reader equilibrated to 37˚C and incubated for 10 min. Following incubation, the plate was ejected and using a 1–10 µL multichannel pipette, 5 µL of DTUN (12 mM) in ethanol was added to the wells and manually mixed again using a 100–300 µL multichannel pipette (set to 250 µL). The microplate was inserted and vigorously shaken for 1 min followed by a 3.5 min rest. Fluorescence was then recorded (λ_ex_ = 488 nm; λ_em_ = 518 nm; gain = 60) every minute for 8 h.

### Preparation of seratrodast hydroquinone

Seratrodrast (25 mg, 0.071 mmol) was dissolved in dry DCM (2.5 mL) in a round-bottomed flask equipped with a magnetic stir bar and sparged with argon for 5 min. Pd/C (12.5 mg, 10 wt% loading on activated carbon support) was added under argon and the flask was stoppered with a rubber septum. The atmosphere was replaced with hydrogen gas and stirred for two days at room temperature. Upon consumption of the quinone, the reaction mixture was filtered over celite and eluted using DCM. Solvent was removed under a stream of argon (to limit oxidation of the hydroquinone) to afford an off-white solid which was subsequently dried under high vacuum to afford the hydroquinone in quantitative yield. ^1^H NMR (400 MHz; DMSO-*d*_6_): ∂ 7.20 (d, *J* = 4.4 Hz, 4H), 7.12–7.05 (m, 1H), 4.25 (t, *J* = 7.8 Hz, 1H), 2.16 (t, *J* = 1.1 Hz, 3H), 2.00 (s, 3H), 1.93 (d, *J* = 1.1 Hz, 3H), 1.89 (d, *J* = 1.1 Hz, 3H), 1.50–1.42 (m, 2H), 1.33–1.14 (m, 4H).

### Cell-free iron-chelating assay

To determine the capacity of seratrodast to chelate ferrous iron (Fe^2+^), the Ferrous Iron-Chelating (FIC) Assay Kit (amsbio) was used according to the manufacturer’s instructions: DMSO (negative control), EDTA (positive control) or seratrodast were diluted in a provided iron(II)sulfate solution before ferrozine was added to start the formation of a colored complex. After incubation at room temperature for 10 min, samples were transferred in triplicates into a clear 96-well plate and absorbance at 562 nm was measured in an EnVision 2104 Multilabel plate reader (PerkinElmer).

### TBARS assay

Two million HT-1080 cells were seeded into 150 mm dishes and grown for 48 h. Ferroptosis was induced with RSL3 for 2.5 h and cells were co-treated with ferrostatin-1 or seratrodast. Cells were detached by 0.05% trypsin-EDTA (Thermo Fisher Scientific) and cell number was adjusted to the sample with the lowest cell count. For measuring MDA levels, TBARS (TCA Method) Assay Kit (Cayman Chemical, 700870) was used according to manufacturer’s instruction. Fluorescence was detected at 530 nm/550 nm in an EnVision 2104 Multilabel plate reader (PerkinElmer).

### Flow cytometry

Cells were grown in 6-well plates (200,000 cells per well) and incubated for 24 h. For staining with BODIPY 581/591 C11 (Thermo Fisher Scientific), cells were treated with RSL3, and ferrostatin-1 or seratrodast for 2 h, then C11-BODIPY was added into the wells to a final concentration of 2 µM. After 30 min incubation, cells were detached using trypsin and washed two times with PBS. Between the wash steps, cells were centrifuged at 500 × *g* for 5 min. After the final washing step, cell pellets were resuspended in 300 µl PBS and 10,000 events per condition were analyzed in the BL-1 channel of an Attune acoustic flow cytometer (Applied Biosystems).

For detection of 4-Hydroxynonenal (4-HNE), HT-1080 were induced with RSL3, and co-treated with ferrostatin-1 or seratrodast for 2 h before they were detached using trypsin. Cell pellets were incubated in 10% normal goat serum (Thermo Fisher Scientific) for 30 min on ice. Cells were then incubated with anti-4-HNE antibody (1:50 dilution in 1% BSA in PBS, ab46545, Abcam) for 1 h on ice. After three washing steps with PBS, cells were resuspended in anti-rabbit Alexa 488 antibody (1:200 dilution in 1% BSA in PBS, A32731, Thermo Fisher Scientific) and incubated for 30 min on ice. For flow cytometry, cells were collected in 300 µl PBS and 10,000 events per condition were analyzed in an Attune acoustic flow cytometer (Applied Biosystems), using the BL-1 channel.

For live/dead discrimination with propidium iodide, HT-1080 were co-treated with RSL3, ferrostatin-1 or seratrodast for 2.5 h before they were detached with trypsin. Cell pellets were washed with PBS twice, resuspended and 1 µg/ml propidium iodide was added. 10,000 events per condition were measured in an Attune acoustic flow cytometer (Applied Biosystems), using the BL-3 channel. For ferroptosis quantification in CD10-135 cells, these were treated with either DMSO, 1.13 µM RSL3, 1.13 µM RSL3 and 1 µM Fer-1, or 1.1.3 µM RSL3 and 6 µM seratrodast for 6 h. After harvesting, pellets were washed twice in PBS and stained with 5 μl of 7-AAD (BD Biosciences) and 5 μl of annexin-V-FITC (BD Biosciences) added to 100 μl annexin-V binding buffer (BD Biosciences). After 15 min, cells were recorded on an LSRII with the FACS Diva 6.1.1 software (BD Biosciences). FlowJo™ v10.8.1 (BD Life Sciences) was used for creating histograms and analyzing fluorescence intensity.

### SDS-PAGE and western blot

HT-1080 were seeded in a 6-well plate at a density of 200,000 cells per well, incubated for 24 h and treated with 6 µM Seratrodast for 6 h. As a control, cells were treated with DMSO. Cells were harvested using a cell scraper and 2× RotiLoad (Roth), before they were sonicated (Sonifier U9200S, Hielscher Ultrasonics) and cooked for 5 min at 95 °C. Lysates were run on a precast NuPAGE 4-12% Bis-Tris gel (Invitrogen) and blotted onto a PVDF membrane via a semi-dry transfer system. After blocking in 5% milk in TBS-Tween, membranes were incubated over night at 4 °C in primary antibody (mouse anti-β-Actin (C4), sc-47778, Santa Cruz Biotechnology and rabbit anti-GPX4 antibody, ab125066, Abcam). Antibodies were diluted 1:1000 (anti-GPX4) or 1:500 (anti-β-Actin) in 2.5% milk in TBS-Tween. Membranes were washed in TBS-Tween three times before incubation in secondary antibody (goat anti-rabbit IgG (H + L) HRP conjugate, W4011; goat anti-mouse IgG (H + L) HRP conjugate, W4021, Promega) for 1 h at room temperature. Secondary antibodies were diluted 1:2500 in 2.5% milk in TBS-Tween. After three more washing steps with TBS-Tween, chemiluminescence was detected using the Western Lightning ECL Pro kit (Perkin Elmer) in a Chemostar ECL imaging system (Intas). Bands were quantified in ImageJ and normalized to β-Actin signal. Uncropped scans are shown in Supplementary Fig. 5.

### Crystal-violet staining

HT-1080 cells were seeded into a 6-well plate at a density of 200,000 cells per well, incubated for 24 h and treated with 200 nM RSL3, 2 µM Fer-1 or 6 µM Seratrodast. DMSO was used as a control treatment. After 24 h, medium was removed, cells were washed with MilliQ water and 1.5 ml crystal violet staining solution (0.5% in 20% methanol). Cells were incubated at room temperature for 20 min and rinsed with milliQ water until no residual crystal violet solution was visible. Images were taken with an EVOS FL fluorescence microscope.

### Murine renal ischemia/reperfusion injury

C57Bl/6N mice were purchased from Charles River (Sulzfeld, Germany) at the age of 6 weeks and kept under stable 12-h circles of darkness and light in IVCs fulfilling at least Euronorm type II. Room temperature was automatically maintained between 20 and 24 °C and air humidity between 45 and 65% as documented in daily controls. Mice had access to sterilized standard pellet food (Sniff) and water *ad libidum*. All cages and nestlets were sterilized by autoclaving before use.

Ten-week-old male mice were co-housed with 2–5 mice/cage in our facility at the Medizinisch-Theoretisches Zentrum (MTZ) at the Medical Faculty of the Technische Universität of Dresden (TU Dresden). A precise protocol for renal ischemia/reperfusion injury has been published [40]. In short, mice received a blinded single dose of either vehicle (4% DMSO in corn oil) or seratrodast (15 mg/kg in vehicle) intraperitoneally with a final volume of 250 µl ice-cold solution 30 min before surgery. Analgesia was achieved by subcutaneous injection of Buprenorphin-HCl (Temgesic, Indivior) followed by anesthesia via isoflurane inhalation. Afterwards, mice were placed in a supine position on a heating plate with a closed-loop temperature regulation system. The abdomen was opened longitudinally followed by visualization and clipping of both renal pedicles (delay between both clips 25–38 s) for 30 min with 100 g pressure serrafines (FST, #18055-03). After release of the clips, reperfusion was confirmed visually, and the abdomen closed via continuous sutures of both layers. To account for fluid losses, 1000 µl pre-warmed PBS (Dulbecco) were injected intraperitoneally before placing the mice at pairs of two back in IVCs. 48 h after surgery, blood was taken via retroorbital puncture and analyzed as part of certified routine analytics in the Institute of Clinical Chemistry of the University Hospital Dresden. After sacrificing the mice, the right kidney was placed in 4% normal buffered formalin for at least 24 h before further processing. The left kidney was snap frozen and preserved for further workup.

For the survival studies, 10-week-old male C57Bl/6N mice received a blinded single dose of either vehicle (4% DMSO in corn oil) or seratrodast (15 mg/kg in vehicle) intraperitoneally with a final volume of 250 µl ice-cold solution 30 min before surgery. Ischemia was induced analogously to the procedure described above, but maintained for 36 min to create a lethal injury. Mice were placed back into IVCs at pairs of two under maintenance conditions. Deterioration was recorded in a blinded manner every 2–4 h, and mice be sacrificed at a predefined human endpoint (“survival”).

### Histology and image acquisition

For histology, kidneys were dehydrated in a graded ethanol series and xylene, and finally embedded in paraffin. Paraffin sections (3–5 μm) were stained with periodic acid–Schiff (PAS) reagent, according to a standard routine protocol. Stained sections were analyzed using an Axio Imager microscope (Zeiss) at ×100, ×200 and ×400 magnification. Micrographs were digitalized using an AxioCam MR R3 FireWire camera and Zen 3.9 software (Zeiss). Pictures were further processed using Affinity Photo 2 software. Renal tubular damage was quantified by an experienced nephropathologist in a double-blind manner on a scale ranging from 0 (unaffected tissue) to 10 (severe organ damage).

For the scoring system, the following parameters were chosen as indicative of morphological damage to the kidney after ischemia-reperfusion injury (IRI): brush border loss, tubule dilatation, tubule degeneration, tubule necrosis, and tubular cast formation. These parameters were evaluated on a scale of 0–10, which ranged from not present (0), mild (1–4), moderate (5 or 6), severe (7 or 8), to very severe (9 or 10).

For TUNEL staining, the Click-iT Plus TUNEL Assay (Invitrogen) was performed according to the manufacturer’s instructions. In short, deparaffinized slides were fixed in 4% PFA for 15 min at 37 °C, and permeabilized with proteinase K. Afterwards, EdUTPs were incorporated for 1 h at 37 °C to detect DNA double-strand breaks. Copper-catalyzed click reaction (30 min at 37 °C) with Alexa Fluor^TM^ picolyl azide was performed using the provided mixtures. After a washing step, slides were incubated with 5 µg/ml DAPI (Invitrogen) for 15 min, and washed three times with deionized water. Pictures were taken using an ApoTome2 (Zeiss) at the Core Facility Cellular Imaging at the TU Dresden (wavelengths: 405 nm and 488 nm, respectively), and analyzed using Zen 3.7 software (Zeiss). To quantify tubular necrosis, a representative area was selected by an experienced nephrologist in a double-blinded manner, and number of TUNEL-positive tubules counted on a ×200 magnification.

### Ethics approval

All mouse experiments were conducted in a strictly blinded manner regarding genotypes and small molecules. All experiments were performed according to German animal protection laws and were approved by German ethics committees and local authorities in Dresden (ethics committee of the TU Dresden and the Landesdirektion Sachsen, respectively).

### Statistics

Statistical analyses were carried out using the GraphPad Prism Software version 9.4. To select the adequate statistical test, we performed a Shapiro–Wilk test for normality and subsequently tested for equal variances using the Brown-Forsythe test. All statistical details, including the number of experiments, are included in the figures and legends. Animal experiments and assessment of renal damage were performed in a strictly double-blinded manner.

## Supplementary information

Supplementary Figures

## Data Availability

Uncropped scans are shown in Supplementary Fig. 5.
