# Supplementary material for: Seratrodast inhibits ferroptosis by suppressing lipid peroxidation
Source: Cell Death Dis. 2024 Nov 22;15(11):853. doi: 10.1038/s41419-024-07251-y (PMC11584883; doi:10.1038/s41419-024-07251-y)
Supplement: Supplementary file 1 — Supplementary Figures [file 41419_2024_7251_MOESM1_ESM.pdf]

Supplementary Figures for:

Seratrodast inhibits ferroptosis by suppressing lipid peroxidation

Juliane Tschuck[1,§], Wulf Tonnus[2,§], Shubhangi Gavali[2,3], Andrea Kolak[1], Melodie Mallais[4], Francesca Maremonti[2,3], Mami Sato[5], Ina Rothenaigner[1], José Pedro Friedmann Angeli[5], Derek A. Pratt[4], Andreas Linkermann[2,3,6], and Kamyar Hadian[1,*]

[1] Research Unit Signaling and Translation, Helmholtz Zentrum München, Neuherberg, Germany

[2] Division of Nephrology, Department of Internal Medicine 3, University Hospital Carl Gustav Carus at the Technische Universität Dresden, Dresden, Germany

[3] Department of Medicine V, University Medical Centre Mannheim, University of Heidelberg, Mannheim, Germany

[4] Department of Chemistry and Biomolecular Sciences, University of Ottawa, Ottawa, ON K1N 6N5, Canada

[5] Rudolf Virchow Center for Integrative and Translational Bioimaging, Chair of Translational Cell Biology, University of Würzburg, Würzburg, Germany

[6] Division of Nephrology, Department of Medicine, Albert Einstein College of Medicine, Bronx, NY, USA

[§] These authors contributed equally

* Corresponding author: Kamyar Hadian, kamyar.hadian@helmholtz-munich.de

Supplementary Figure 1

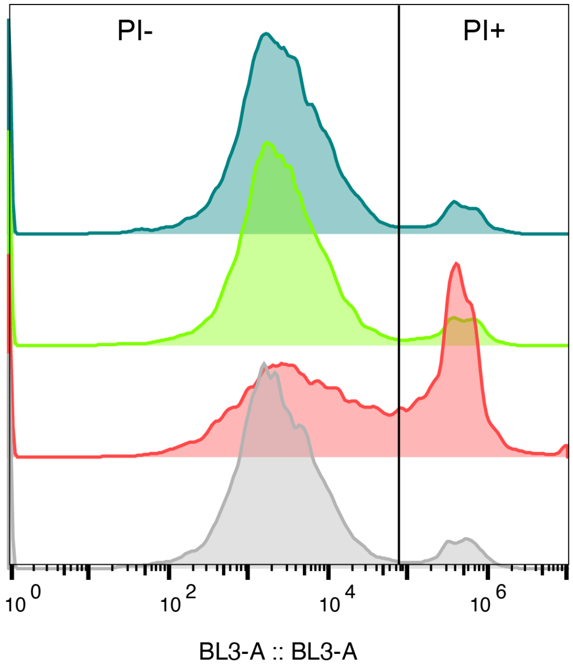

Supplementary Figure 1:

Seratrodast rescues HT-1080 from RSL3-induced cell death. Representative histogram of HT-1080 cotreated with 200 nM RSL3, 6 µM seratrodast or 2 µM ferrostatin-1 for 2.5 h, stained with 1µg/ml propidium iodide and analyzed via flow cytometry. Grey = DMSO, red = RSL3, light green = RSL3 + Fer-1, dark green = RSL3 + seratrodast. The gate was set between the two peaks to discriminate between PI negative (live) and PI positive (dead) cells, percentages of PI+ cells were statistically analyzed.

Supplementary Figure 2

A

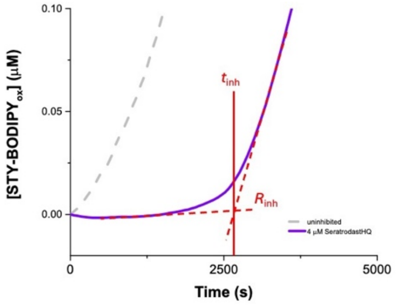

$$[1]\quad n = \frac{t_{inh} \times R_i}{[RTA]}$$

$$[2]\quad k_{inh} = \frac{k_{STY\text{-}BODIPY}\,[STY\text{-}BODIPY]\,R_i}{R_{inh}\,[RTA]}$$

B

R_i	$(9.8 \pm 0.5) \times 10^{-10}$ M/s		
	t_{inh} (s)	n	k_{inh} ($\times 10^4$ $M^{-1}s^{-1}$)
SeratrodastHQ	2984 ± 130	0.5 ± 0.02	4.2 ± 0.2
Fer-1	12342 ± 793	3.0 ± 0.02	6.6 ± 0.1
PMC	8178 ± 471	(2.0)	3.7 ± 0.5

Supplementary Figure 2:

A Representative data for the seratrodast hydroquinone-inhibited co-autoxidation of STY-BODIPY and egg phosphatidylcholine. The radical trapping stoichiometry (n) and inhibition rate constant (k_{inh}) can be determined directly from the duration of the inhibited period (t_{inh}) and the initial rate of the inhibited portion of the autoxidation using equations 1 and 2, respectively. The data are fit from ~500 seconds, as an equilibration period is required for the rate of STY-BODIPY oxidation to reach a constant value. **B** Averages of n and k_{inh} values determined as in A from three independent autoxidations inhibited by seratrodast hydroquinone, ferrotstatin-1 and PMC (each at 4 μM).

Supplementary Figure 3

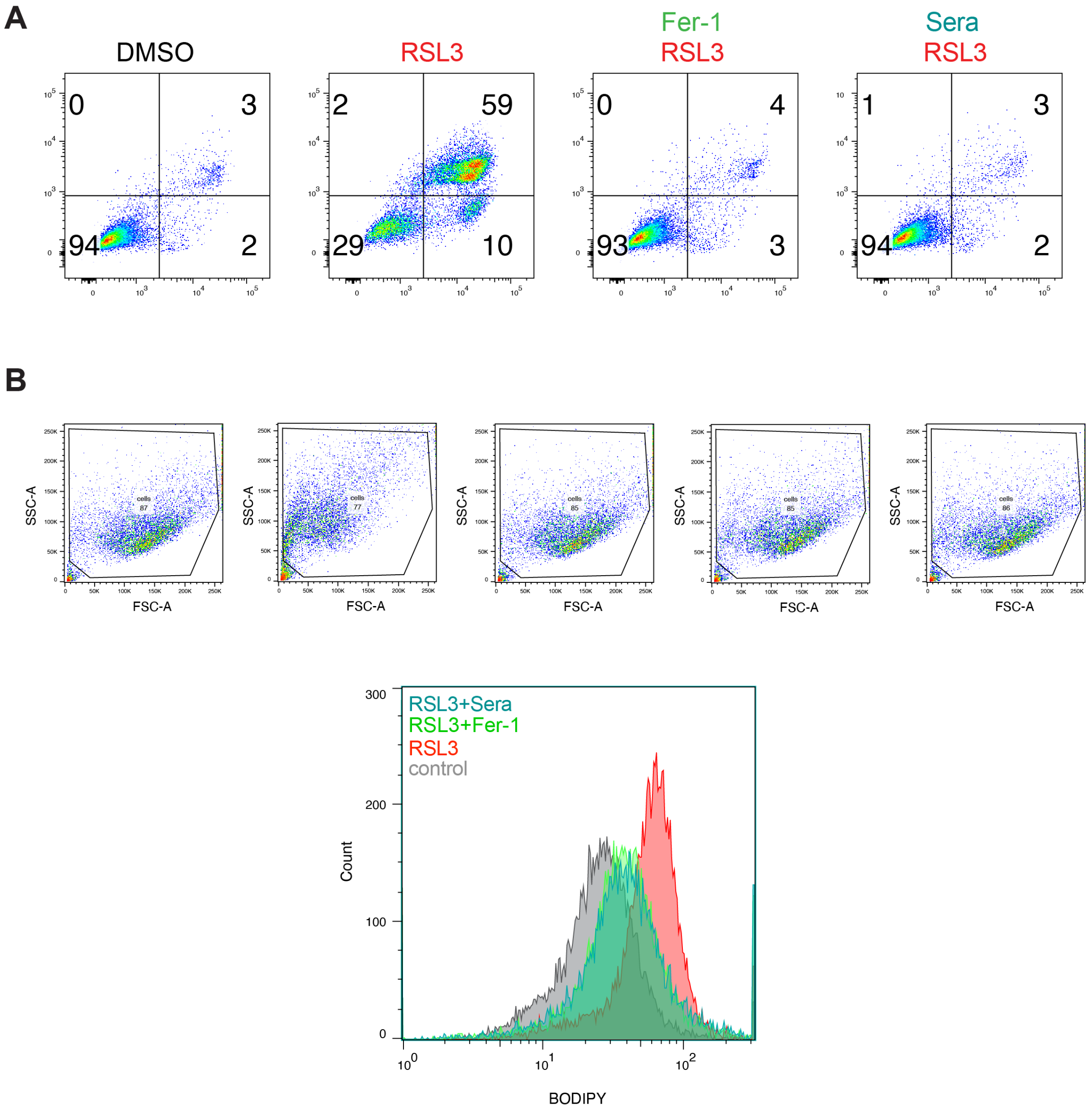

Supplementary Figure 3:

A Annexin V/7-AAD staining of CD10-135 cells shows a rescue of RSL3-induced cell death in seratrodast-treated cells. 1.13 µM RSL3, 1 µM Fer-1, 6 µM seratrodast for 6h. Scatter plots show percentages of cells in the respective quadrants. **B** Seratrodast treatment reduces lipid peroxidation in C11-BODIPY-stained CD10-135 cells. 1.13 µM RSL3, 1 µM Fer-1, 6 µM seratrodast for 4 h. Data shown are representative scatter plots and histograms of independent flow cytometry experiments.

Supplementary Figure 4

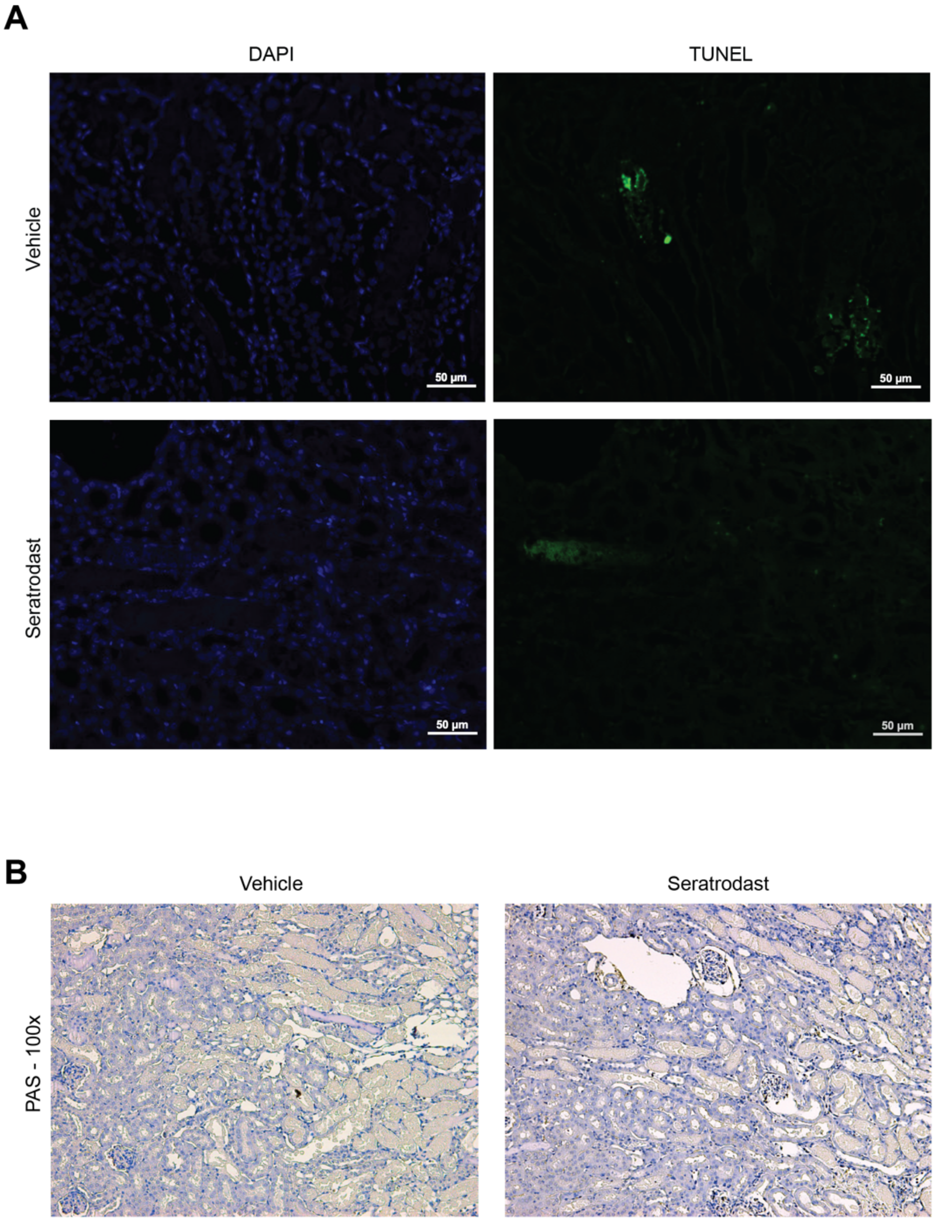

Supplementary Figure 4:

A Representative images of TUNEL-stained murine kidneys after IRI-induction. Treatment with seratrodast alleviates acute tubular necrosis. DAPI staining was performed for normalization of cell count. Images were taken with 200x magnification. **B** Representative images of Periodic acid-Schiff staining (PAS) in IRI-induced mice for visual assessment of kidney damage. Seratrodast treatment improves the kidney damage score. Images were taken at 100x magnification.

Supplementary Figure 5

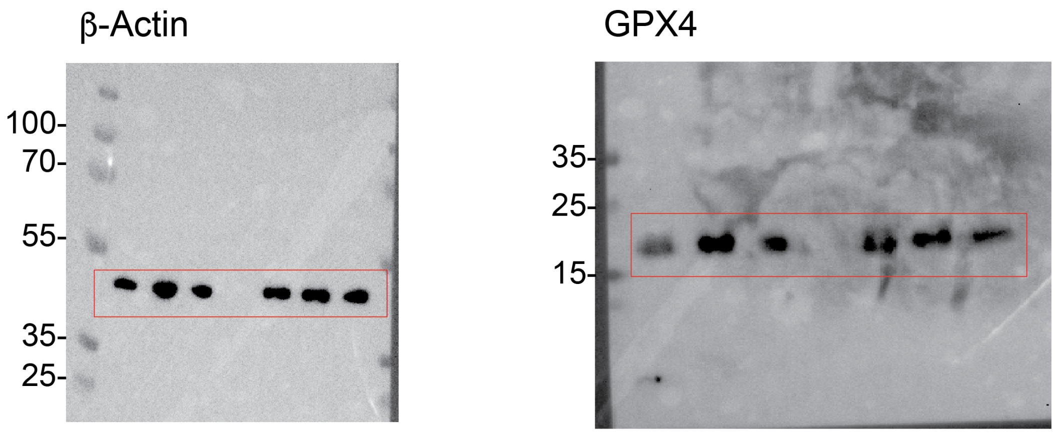

Supplementary Figure 5: Full scans of the Western Blots in Fig. 2C
